# Preparing for cell culture scale-out: establishing parity of bioreactor- and flask-expanded mesenchymal stromal cell cultures

**DOI:** 10.1186/s12967-019-1989-x

**Published:** 2019-07-24

**Authors:** Ruud Das, Rens Roosloot, Melissa van Pel, Koen Schepers, Marijn Driessen, Willem E. Fibbe, Joost Dick de Bruijn, Helene Roelofs

**Affiliations:** 1 Scinus Cell Expansion BV, Professor Bronkhorstlaan 10, Building 48, 3723 MB Bilthoven, The Netherlands; 2 Leiden University Medical Centre, Albinusdreef 2, 2333 ZA Leiden, The Netherlands; 3 Twente University, Drienerlolaan 5, 7522 NB Enschede, The Netherlands; 4 Queen Mary, University of London, Mile End Road, London, E1 4NS UK

**Keywords:** Bioreactor, Mesenchymal stromal cells, Cell therapy

## Abstract

**Background:**

Cell-based therapies have the potential to become treatment options for many diseases, but efficient scale-out of these therapies has proven to be a major hurdle. Bioreactors can be used to overcome this hurdle, but changing the culture method can introduce unwanted changes to the cell product. Therefore, it is important to establish parity between products generated using traditional methods versus those generated using a bioreactor.

**Methods:**

Mesenchymal stromal cells (MSCs) are cultured in parallel using either traditional culture flasks, spinner vessels or a new bioreactor system. To investigate parity between the cells obtained from different methods, harvested cells are compared in terms of yield, phenotype and functionality.

**Results:**

Bioreactor-based expansion yielded high cell numbers (222–510 million cells). Highest cell expansion was observed upon culture in flasks [average 5.0 population doublings (PDL)], followed by bioreactor (4.0 PDL) and spinner flasks (3.3 PDL). Flow cytometry confirmed MSC identity (CD73^+^, CD90^+^ and CD105^+^) and lack of contaminating hematopoietic cell populations. Cultured MSCs did not display genetic aberrations and no difference in differentiation and immunomodulatory capacity was observed between culture conditions. The response to IFNγ stimulation was similar for cells obtained from all culture conditions, as was the capacity to inhibit T cell proliferation.

**Conclusions:**

The new bioreactor technology can be used to culture large amounts of cells with characteristics equivalent to those cultured using traditional, flask based, methods.

**Electronic supplementary material:**

The online version of this article (10.1186/s12967-019-1989-x) contains supplementary material, which is available to authorized users.

## Background

Cell-based therapies have the potential to become treatment options for many diseases [1], but efficient scale-out of these therapies has proven to be a major hurdle [2, 3]. The production of adherent cells for therapeutic purposes is particularly difficult, due to the need for a large surface area to expand cells to clinically relevant numbers. Traditionally, adherent cell expansion for clinical application involves cell culture in large numbers of flasks in incubators installed in cleanroom facilities. This method requires heavy operator involvement as the culture needs to be seeded, refreshed, passaged and harvested individually and manually. The high costs of cell production in flasks poses a risk to the commercial viability of these cell-based therapies by jeopardizing the positive outcome of a cost–benefit assessment, which is necessary for therapy reimbursement [4]. Consequently, the need for more efficient culture methods that would enable a widespread deployment of cell therapies is well established [5].

Several alternatives that aim to address the specific problems associated with flask culture have come to market. These include stacked and multi-layered flask systems, as well as various bioreactor systems.

The use of bioreactors reduces the amount of labour and allows more space-effective production. Moreover, the use of closed systems enables cell therapy medicinal product (CTMP) manufacturing in environments with less stringent good manufacturing practise (GMP) classification thereby reducing production costs, compared to expansion using flasks. However, changing the culture methods can introduce changes to the characteristics and functionality of the cell products [6], as the properties of the cell product may change upon minor changes in cell manipulation. Therefore, it is crucial to investigate whether proposed new culture methods result in a product that is comparable in terms of identity, safety and potency [7].

Mesenchymal stromal cells (MSCs) are currently investigated for clinical application in numerous trials rendering this adherent cell population highly relevant for cell therapy manufacturing using bioreactors. Here we investigate the possibility to efficiently generate MSCs using a novel, closed bioreactor system (Scinus), designed for automated cell expansion. We compared the characteristics of MSC populations produced with three different culture methods. We expanded MSCs on dissolvable microcarriers in the Scinus and in spinner flasks—which are a down-scaled model for bioreactor culture- and compared this to the traditional flask-based MSC production method. The comparison included MSC expansion potential and key attributes of the resulting cell populations. The expansion potential was assessed in MSC cultures where the MSCs were expanded to therapeutic relevant numbers (typically multiple infusions at a dose range of 1–2 millions of cells per kg of recipient’s body weight, amounting to several hundred millions of cells). MSCs have been attributed many functional properties [8]. They can modulate immune responses [9], secrete various factors contributing to tissue repair [10] and can be differentiated towards relevant cell types for tissue replacement [11]. Since the immunomodulatory properties of MSCs are widely recognised and are frequently included in the proposed mechanisms of action for various therapeutic effects [12], we focus on these immunomodulatory properties and the responsiveness to inflammatory cytokines as indicators for the therapeutic potential of the MSCs.

For one CTMP manufacturing process to be replaced by another, parity between the cell products from both processes needs to be established [7]. We demonstrate that these bioreactor-expanded MSC populations are phenotypically similar to flask- and spinner-expanded MSC populations and are functionally equivalent. Expansion in the bioreactor yields clinically relevant cell numbers.

## Methods

Bone marrow processing, MSC pre-culture, parallel expansion in flasks, spinners and in the Scinus bioreactor, cell harvest and microscopic inspection of the expanded cells was performed at Scinus Cell Expansion BV. All cellular and molecular analyses of the resulting MSC populations were independently performed at the Leiden University Medical Centre.

### The Scinus cell expansion bioreactor

**Fig. 1:**
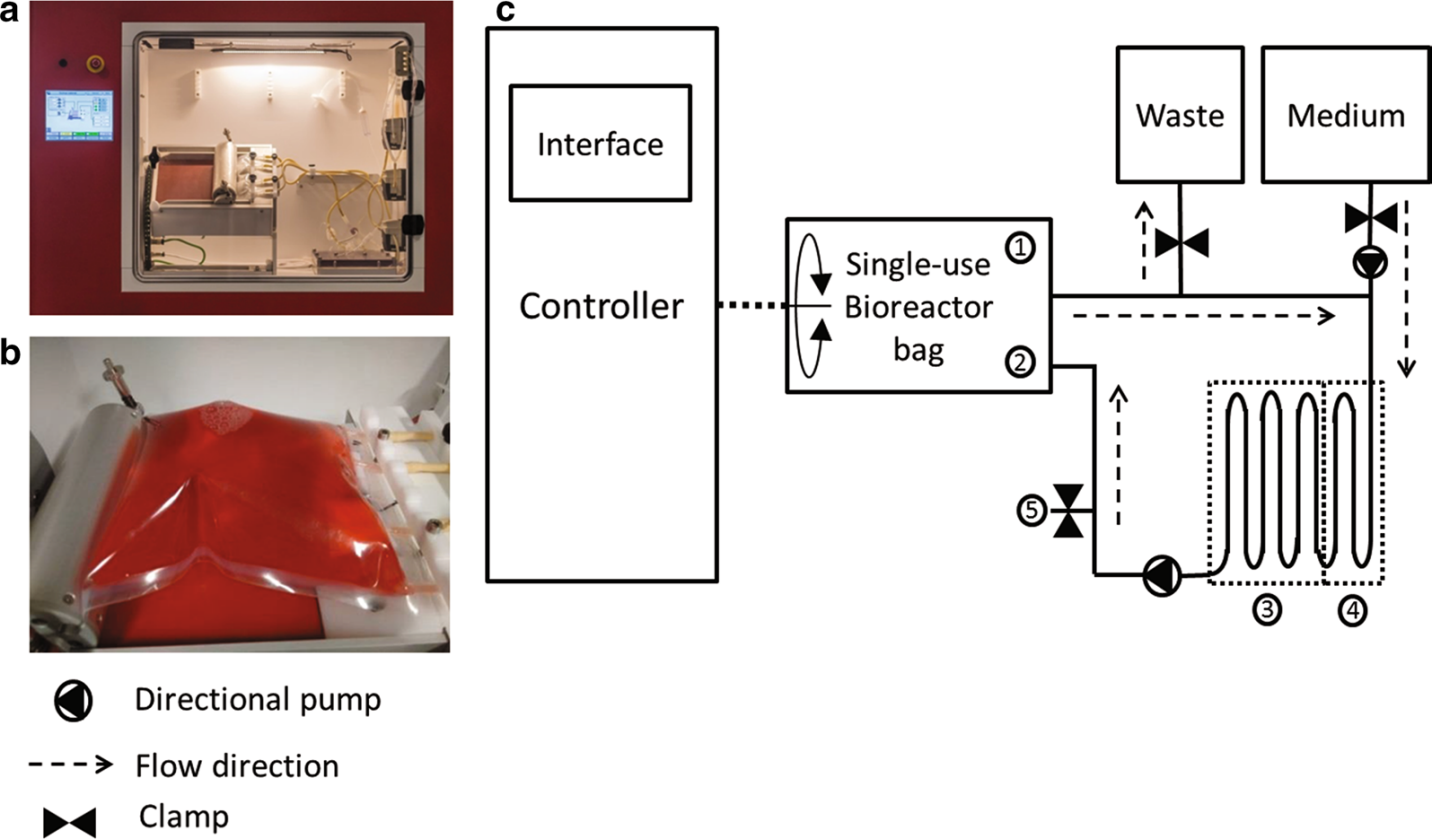
Overview of the Scinus bioreactor system. **a** Scinus Bioreactor cabinet, **b** single-use bioreactor bag and **c** a schematic representation of the Scinus bioreactor system. 1: pH sensor, 2: DO sensor, 3: DO control section of the oxygenator, 4: pH control section of the oxygenator, 5: sample port

The Scinus Cell Expansion system was developed for the controlled expansion of cells. For the expansion of adherent cells, such as MSCs, microcarriers are used. The system consists of a single-use cell culture bag which is placed on a platform inside the hardware enclosure (Fig. 1a, b). The platform can rotate along its longitudinal axis at predefined speeds and intervals, thus maintaining a homogeneous cell suspension. The bag is coupled to a tubing system for the addition of fresh medium and the removal of waste medium (Fig. 1c). The medium is perfused through an oxygenation system that controls the dissolved oxygen (DO) and pH as measured by chemical optical sensors (PreSens GmbH, Regensburg, Germany) that are incorporated in the bag. The temperature of the culture environment is controlled by the hardware enclosure. The system maintains a closed environment, and the addition (e.g. of fresh medium) and removal (e.g. sampling of cell suspensions or spent medium) is performed through tube welding. Furthermore, the Scinus features an option to expand the volume of the bioreactor bag, ranging from 100 to 1400 mL.

### Isolation and pre-culture of bone marrow-derived MSCs

Bone marrow biopsies were obtained from patients undergoing total hip replacement surgery. These biopsies were taken at the St. Antonius hospital, Utrecht, The Netherlands, after written informed consent was obtained. Ethical approval for this study was granted by the local medical ethics committee (study reference 41885.100.12 R12.037/BOBI).

The bone marrow aspirate was filtered through a 100 μm cell strainer (Merck/Millipore) and diluted 1:1 with phosphate buffered saline (PBS, Westburg). Next, mononuclear cells (MNC) were obtained by density centrifugation using Lymphoprep density gradient medium according to the manufacturer’s protocol (Axis Shield) and seeded at approximately 160,000 cells/cm^2^ in T-flasks (Greiner) in hMSC medium. The hMSC medium consisted of alpha-MEM (Westburg), 15% fetal bovine serum (FBS, Invitrogen), 1 ng/mL basic fibroblast growth factor (bFGF, Serotec), 2 mM GlutaMAX-I (Invitrogen), 0.2 mM ascorbic acid-2-phosphate (Sigma-Aldrich) and 100 U/100 μL penicillin/streptomycin (Invitrogen). The medium was refreshed every 2–3 days. Cells were harvested (passage 1) at 70–80% confluency, resuspended in freezing medium [hMSC medium with 30% FBS and 10% dimethylsulfoxide (DMSO)] and stored in liquid nitrogen until further use.

Cells were thawed, seeded at 400 cells/cm^2^ in T-flasks and cultured in hMSC medium for one passage. The cells were harvested (passage 2) at 70–80% confluency. After harvesting, the cells were used for parallel cultures in either T-flasks (Flask), spinner (Spinner) or the Scinus Cell Expansion system (Scinus).

### Microcarrier suspension

For Scinus- and spinner-expansion, denatured collagen-coated, dissolvable microcarriers (DMC) were used (Corning Life Sciences). These microcarriers have a surface area of 5000 cm^2^/g. They were prepared according to the manufacturer’s instructions and stored as a stock solution at 5.0 g/L in hMSC medium.

### Parallel expansion procedures

All expansion procedures were maintained for a total of 6 days, after which cells were harvested for various analyses.

#### Flask expansion

Monolayer cultures were seeded at 400 cells/cm^2^ in T-flasks in hMSC expansion medium. The flasks were kept in a standard incubator at 37 °C, 5% CO_2_ and 100% humidity. The medium was refreshed every 2–3 days.

#### Spinner expansion

Spinners (BellCo) were prepared with 50 mL of microcarrier suspension and inoculated with 1.25 million cells (25,000 cells/mL, 1000 cells/cm^2^). Overnight seeding and subsequent expansion was performed with 9 cycli of continuous agitation at 35 RPM for 7 h, followed by a 1 h static interval to facilitate attachment of the cells. After 3 days, the concentration of microcarriers was increased to 10 g/L and the static interval was removed. Spinners were kept in a standard incubator at 37 °C, 5% CO_2_ and 100% humidity. The medium was refreshed every 2–3 days.

#### Scinus expansion

One day prior to cell seeding, a Scinus Cell Expansion system (Scinus Cell Expansion BV, Bilthoven, The Netherlands) was prepared as follows. A single-use bioreactor bag was filled with approximately 800 mL of microcarrier suspension. The culture environment was primed overnight to reach setpoints of 75% DO, pH 7.3, and a temperature of 37 °C. After priming, a total of 20 million MSCs was inoculated (25,000 cells/mL, 1000 cells/cm^2^). A homogeneous suspension was maintained by rocking the bioreactor bag for 7 h followed by a 1 h static interval. The settings for agitations are shown in Table 1. During the first 24 h, the system was not perfused and only temperature was controlled. Thereafter, perfusion was started at 2 mL/min in order to maintain setpoints for DO and pH. After 3 days, the concentration of microcarriers was increased to 10 g/L and the static interval was removed.

**Table 1:** Settings for the dynamic agitation platform of the Scinus cell expansion system

Parameter	Dimension	Setting	Description
Max angle	Degrees (°)	180	Maximum rotation angle of the platform along its longitudinal axis. 180° results in a completely vertical position on both sides
Speed	(°/second)	90	Speed of rotation of the platform along its longitudinal axis
Acceleration	(°/second^2^)	90	Acceleration of the platform until the designated speed is achieved
Deceleration	(°/second^2^)	90	Deceleration of the platform until the speed is 0°/s
Vertical pause	(seconds)	10	Vertical hold time when the rotation has achieved the maximum angle, after which the platform rotates in the opposite direction. Used to let microcarriers settle and minimize shear
Horizontal pause	(time, e.g. hours)	One hour pause after every 7 h of agitation	Hold time in horizontal (rest) position, used to optimise bead-to-bead transfer

#### Harvest

Cells were harvested using 1× TrypLE (Invitrogen) for 15 min. The microcarriers in spinner and Scinus cultures were subsequently dissolved for 15 min using a dedicated harvest solution consisting of PBS with pectinase (Sigma) and EDTA (0.5 M pH 8.0, Corning Life Sciences) at final concentrations of 100 U/mL and 10 mM respectively. Dissolution resulted in a single-cell suspension. The cells were washed in PBS and stored at − 80 °C in freezing medium (hMSC medium with 30% FBS and 10% DMSO) until analysis in various assays.

### Analyses

#### Expansion

Microscopic inspection was performed daily to monitor the morphology of cells in T-flasks and on microcarriers. In addition, cell counts were performed at least on day 4 and 6 for flask and spinner cultures, and every 1–2 days for Scinus cultures. Population doubling levels at harvest (PDL_harvest_) were calculated according to the following formula:

$$PDL_{harvest} = \frac{{\ln (C_{harvest} /C_{seed} )}}{{{ \ln }\left( 2 \right)}},$$

where C_harvest_ is the total cell number at harvest, C_seed_ is the number of cells seeded.

#### Surface marker expression

Expression of surface markers was determined on cell samples that were cryopreserved on day 6. Cells were thawed and evaluated for the following markers: CD73, CD105, CD90, CD45 and CD3. Data acquisition was performed on a FACSCanto using Diva-software (BD Biosciences, Erebodegem, Belgium). The data was analysed using Flow-jo software.

#### Karyotyping

The cells were seeded in culture flasks to stimulate proliferation. After treatment with colcemid (4-6 h at 0.15 μg/mL) the cells were detached using trypsin and concentrated by centrifugation. The cells were treated with hypotonic solution (75 mM KCl, 3 min), concentrated again and fixed with 10 volumes fixative 1 [methanol-acetic acid 9:1 (v/v)]. The cells were concentrated and subsequently fixed with 10 volumes fixative 2 [methanol-acetic acid 3:1 (v/v)]. The fixed cell suspension was concentrated once more after which the cells were dropped onto microscope slides. The slides were allowed to dry and aged overnight at 60 °C. The slides were re-hydrated in 2× SSC and subsequently in PBS, treated with trypsin (0.07%, 3 min) and finally stained at room temperature for 3–4 min according to Giemsa-Leishman (G-banding) using 1 ml Giemsa (Merck) and 3 mL Leishman stain (Merck) in 60 ml Gurr buffer (Gibco). The slides were dried and mounted in Eukitt^®^.

#### Stimulation assays

MSCs generated by all three expansion methods were thawed, subcultured in flasks at a concentration of 4000 cells per cm^2^ and harvested when at least 80% confluence was reached. One day prior to stimulation 50,000 MSC/well were seeded in hMSC medium in a 12-well plate (Corning, United States). MSCs were stimulated for a total of 48 h with IFNγ (Peprotech, London, UK) at a concentration of 10 ng/mL. At 30 h into the MSC-stimulation, Monensin protein transport inhibitor (BD Biosciences, Breda, the Netherlands) was added according manufacturer’s instructions and stimulation continued for the remaining 18 h. Next, expression of indoleamine 2,3-dioxygenase (IDO) and CD274 was evaluated using flow cytometry. Data acquisition was performed on a FACSCanto using Diva-software (BD Biosciences, Erebodegem, Belgium). The data was analysed using Flow-jo software.

#### Inhibition of T cell proliferation

The capacity of MSCs to inhibit T cell proliferation was tested in a co-culture of MSCs with peripheral blood mononuclear cells (PBMCs). MSCs were plated in graded doses in a 96-well V-bottomed plate in RPMI 1640 supplemented with 10% FBS and penicillin/streptomycin (all from Thermo Fisher Scientific). PBMCs (1.0 × 10^5^/well) were added and stimulated with human T-activator CD3/CD28 Dynabeads (Thermo Fisher Scientific) in a bead:PBMC ratio of 1:5. The co-cultures were incubated for 6 days at 37 °C, where ^3^H-thymidine was added for the last 16 h. ^3^H-Thymidine incorporation was measured on an oscillation counter (MSD Nordian) and data was expressed as a percentage of the positive control (no MSC). The heatmap and the Pearson correlation average-linkage analysis-based hierarchical clustering was performed with the TM4 MeV Stand-Alone Client [13].

#### Differentiation assays

Tri-lineage differentiation was assessed for cells seeded directly from cryopreservation. Adipogenic differentiation was performed using the MesenCult Adipogenic Differentiation Kit (Stemcell Technologies) according to the manufacturer’s instructions. Similarly, chondrogenic differentiation was done using the MesenCult Chondrogenic Differentiation Kit (Stemcell Technologies) according to the manufacturer’s instructions. The osteogenic differentiation protocol consisted of 25–28 day culture on hMSC medium with the addition of β-glycerolphosphate (0.01 M) and dexamethasone (10^−8^M). For adipogenic differentiation, presence of lipid vacuoles was demonstrated using Oil Red O staining. For chondrogenic differentiation, presence of aggrecan was demonstrated using Alcian Blue staining. For osteogenic differentiation, calcium deposits were stained with Alizarin Red.

#### Statistics

Matched-samples ANOVA was performed to test for difference in CD73, CD90 and CD105 expression. The Friedman test was performed to determine the effect of the expansion method on IFNγ-induced upregulation of CD274 and IDO expression. The Kruskal–Wallis test was performed to determine the effect of donor background.

## Results

### Initiation of bone marrow culture

MSC cultures were initiated from bone marrow aspirates derived from five different donors. Average donor age was 65.4 years (range 57–82 years) with three male and two female donors. Biopsies were small (range 6.1–13.0 mL) and white blood cell counts ranged from 8.5 × 10^6^ to 28.3 × 10^6^ cells/mL. Donor and biopsy information is summarised in Table 2.

**Table 2:** Donor information

Donor ID	Age (years)	Gender	Volume (mL)	White blood cell count (cells/mL)	PDT of second passage (h)
1	62	Male	6.1	22.6 × 10^6^	22.9
2	62	Male	13.0	8.5 × 10^6^	29.9
3	57	Male	6.3	12.1 × 10^6^	21.0
4	82	Female	8.0	28.3 × 10^6^	31.1
5	64	Female	10.5	9.0 × 10^6^	34.6

### Characterisation of flask, spinner and Scinus cultures

#### Cell expansion

**Fig. 2:**
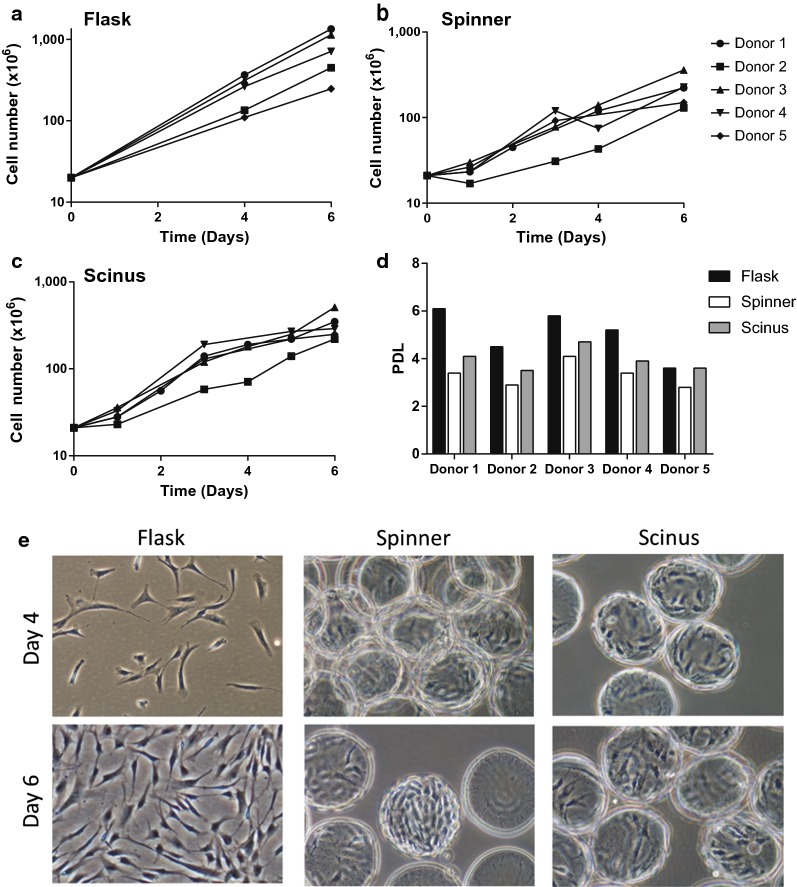
Parallel MSC expansion in **a** flasks, **b** spinner and **c** Scinus cultures for 5 donors. Cell numbers were normalized to a 20 × 10^6^ inoculum of MSCs. **d** Corresponding PDLs for each culture. **e** Visual inspection of the three different culture conditions on day 4 and 6

Each single-donor BM-derived MSC population was parallel-cultured in flasks and on microcarriers in both the Scinus and in a spinner. All cultures were evaluated for cell numbers at multiple time points. To directly compare the cell yields to the Scinus cultures, all cell numbers were normalised to a 20 × 10^6^ inoculum, resulting in a calculated yield of 778 × 10^6^ (average, range 247–1340 × 10^6^) and 218 × 10^6^ MSCs (average, range 130–360 × 10^6^) for flask and spinner cultures, respectively (Fig. 2a, b). On average 324 × 10^6^ cells (range 220–510 × 10^6^) were cultured in 6 days in the Scinus from a 20 × 10^6^ inoculum (Fig. 2c), corresponding to a population doubling level of 4.0 (range 3.5–4.7). The highest PDL was observed for MSCs grown in flasks (average PDL 5.6 ± 1.8; Fig. 2d). Spinner cultures exhibited the lowest expansion (average PDL 3.3 ± 0.5; Fig. 2d).

#### Morphology and confluency

On day 4 and 6 of culture, morphology of the cells and confluency of the cultures was analysed. Cells expanded on microcarriers retained the spindle shaped morphology typically described for monolayer cultures of MSCs (Fig. 2e). Microscopic inspection showed that the cells were able to occupy freshly added microcarriers. On day 6, especially in the spinner cultures, some microcarriers were more densely populated than others and the cells and microcarriers started to aggregate.

#### Surface marker expression

**Fig. 3:**
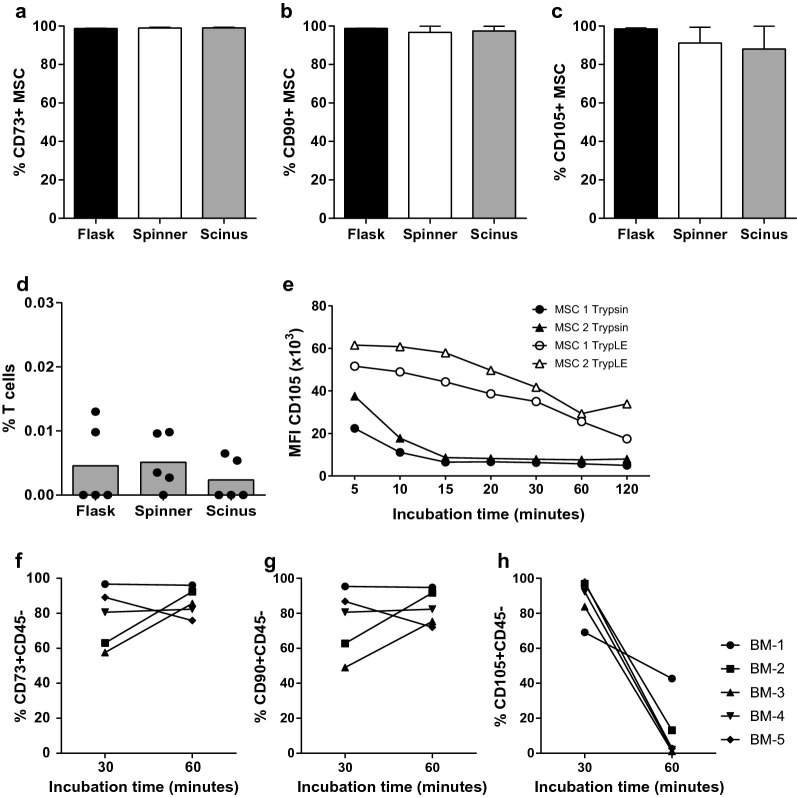
Identity and purity of the cultured MSCs. **a**–**c** Group analysis of expression of CD73, CD90 and CD105 on MSCs harvested from flask, spinner and Scinus cultures. **d** Presence of remaining T cells in the parallel cultures. **e** Influence of incubation time with cell dissociative reagents on CD105 expression. **f**–**h** Comparison of CD73, CD90 and CD105 surface marker expression after 30 and 60 min of incubation with TrypLE

The cells harvested on day 6 were evaluated for cell surface marker expression. The frequency of cells expressing CD73 and CD90 was the same for the flasks (p = 0.401 resp. 0.247), spinner and Scinus cultures (Fig. 3a, b). We observed a lower frequency of CD105 expressing cells in spinner and Scinus cultures, compared to MSCs expanded in flasks (Fig. 3c), although this difference was not statistically significant (p = 0.245). The frequency of CD3^+^ T cells was less than 0.01% of the cultured cells (Fig. 3d) and was similar for the three culture conditions. In none of the three culture conditions B cells, NK-cells, monocytes, macrophages or hematopoietic stem- or progenitor cells could be detected (data not shown).

Since the harvesting procedures for the cells expanded on microbeads take a longer amount of time than the harvesting procedure for the flask-expanded cells, we hypothesized that this difference in downstream processing was the cause of the observed phenotype difference (reduced CD105 expression). Prolonged exposure to pectinase was not considered as a potential cause for the reduced expression, since this enzyme is specific to the degradation of polysaccharides found in plant cell walls. To investigate whether prolonged exposure to the cell dissociative reagent TrypLE may explain the reduced CD105^+^ cell frequency in spinner- and Scinus-cultured MSC, a timing experiment was set up as follows. MSCs were cultured in multiple flasks until 90% confluency and subsequently harvested after various times of incubation with TrypLE (up to 120 min). Incubation with trypsin, a more aggressive cell dissociative reagent commonly used in research, served as a positive control. CD90 and CD73 median fluorescence intensity (MFI), although different for individual MSCs derived from different donor populations, was stable for at least 30 min of incubation in trypsin as well as in TrypLE (unpublished observation). In contrast, CD105 MFI was reduced already after 5 min of incubation in trypsin and reached its lowest levels after 15 min of incubation. Incubation with TrypLE also induced a reduction in CD105 MFI, but this decline was slower and steady over 60–120 min time (Fig. 3e). In the parallel culture experiments, cells were harvested using TrypLE. To investigate whether CD73, CD90 and CD105 expression was affected by TrypLE incubation time, the expression was assessed after 30 and 60 min incubation with TrypLE. The proportion of CD73 and CD90 expressing cells was not decreased following TrypLE incubation, while the proportion of cells expressing CD105 was significantly reduced (p < 0.001; Fig. 3f–h). This indicates that cell dissociative reagents, including TrypLE, can affect MFI levels and that CD105 is more susceptible to this effect than CD73 and CD90. Therefore, the observed phenotypic difference between flask-and Scinus-cultured MSCs is most likely not related to the expansion process, but rather to the downstream processing steps used to collect the cells.

#### Differentiation capacity

**Fig. 4:**
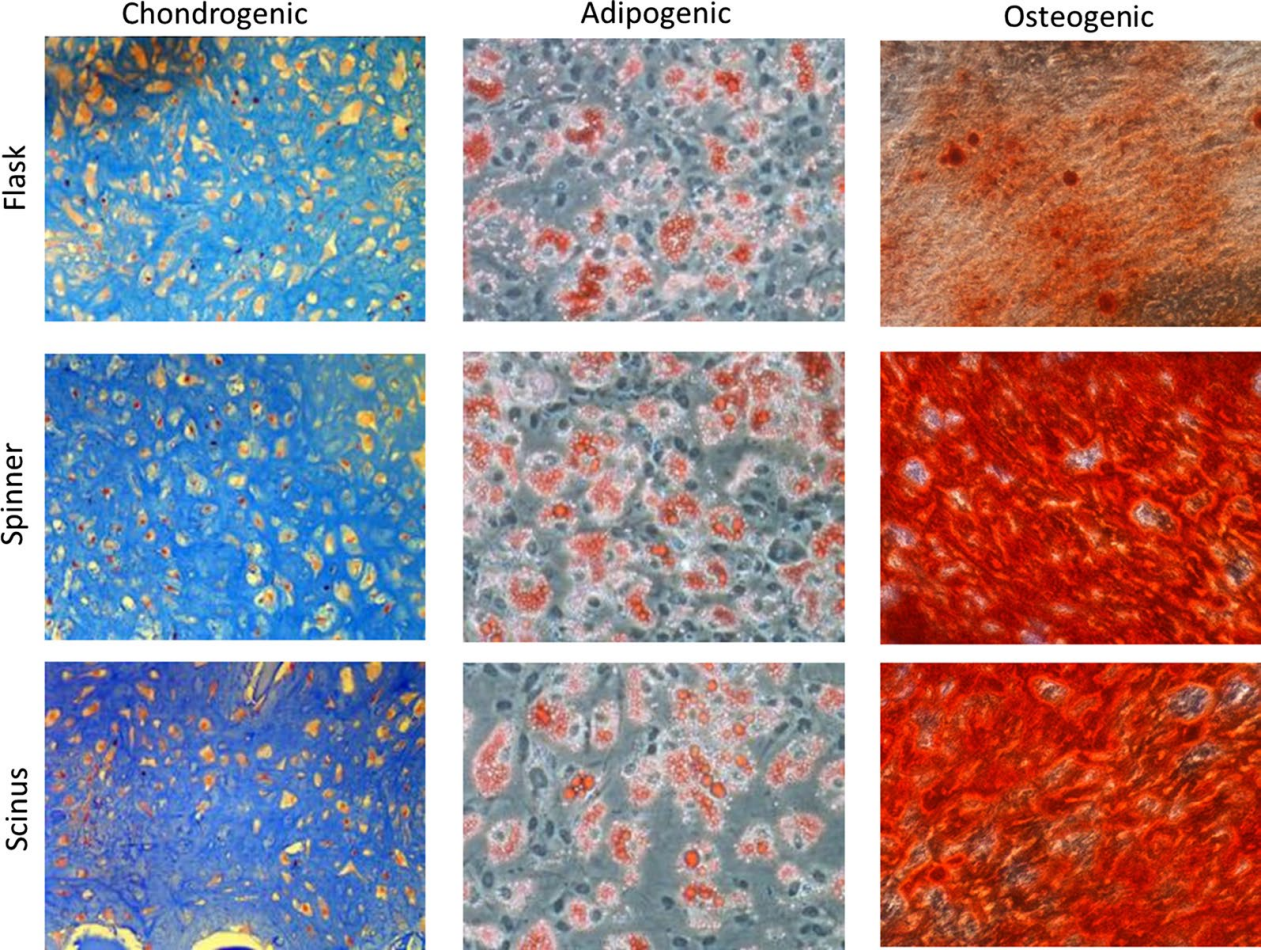
Differentiation assay results for Donor 1. Tri-lineage differentiation is observed for all culture conditions for this donor. Results for all donors are provided in Additional file 1

Differentiation capacity along the three lineages associated with MSC phenotype (osteo-, adipo- and chondrogenic) was similar for all conditions (Donor 1 results shown in Fig. 4). Adipogenic and chondrogenic differentiation was observed for all cells from all different culture methods. Osteogenic differentiation was not observed for all donors, but differentiation capacity was clearly donor dependent, as no qualitative difference was observed between culture conditions using the same donor material (Additional file 1 shows results for all available conditions).

#### Karyotype

The five Scinus-expanded as well as the five flask-expanded cultures were analysed by karyotyping for the presence of genetically aberrant cells. Although the target number of 20 analysable metaphases was not reached in the 10 karyotype analyses performed, no signs of genetic aberration were observed in any of the MSC populations (Table 3).

**Table 3:** Karyotype analysis

Number of metaphases			
Donor ID	Culture method	Analysed	Result
1	Flask	8	Normal male
	Scinus	5	Normal male
2	Flask	17	Normal male
	Scinus	0	None
3	Flask	0	None
	Scinus	15	Normal male
4	Flask	4	Normal female
	Scinus	2	Normal female
5	Flask	10	Normal female
	Scinus	16	Normal female

### No differences in immunomodulatory phenotype and capacity between Scinus-, spinner- or flasks-cultured MSCs

#### Response to cytokine stimulation

**Fig. 5:**
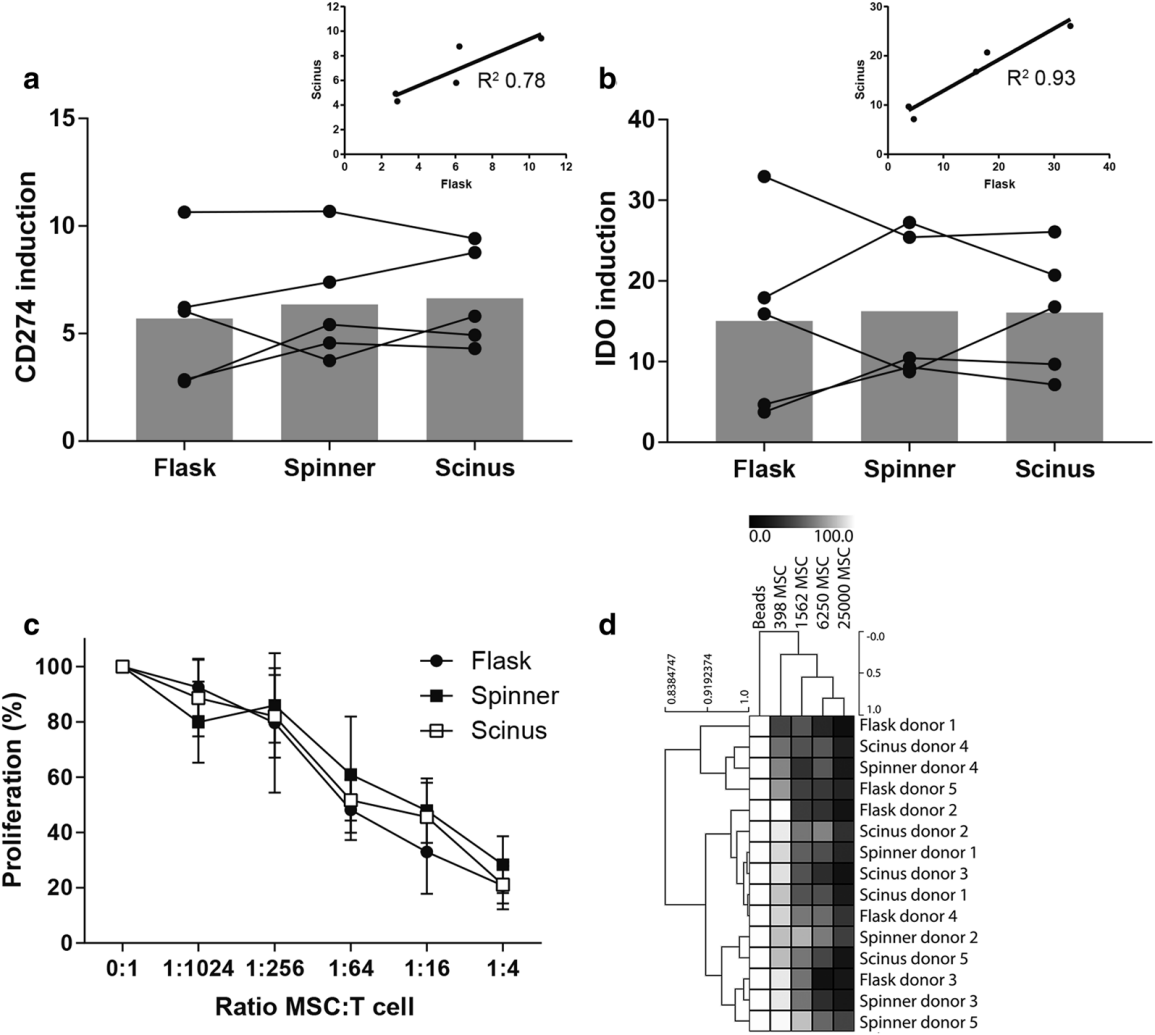
Immunomodulatory capacity of flask-, spinner- and Scinus-cultured MSC. **a**, **b** Induction of CD274 and IDO protein expression in response to pro-inflammatory cytokine IFNγ. Induction is expressed as MFI (stimulated cells)/MFI (unstimulated cells). The Friedman test showed no statistically significant difference in IFNγ-induced upregulation of CD274 and IDO between cells cultured in different culture conditions, χ^2^(2) = 1.600, *p* = 0.449 and χ^2^(2) = 2.333, p = 0.311 respectively. The Kruskal–Wallis H test showed a statistically significant difference in IFNγ-induced upregulation of CD274 and IDO between MSCs grown from the different bone marrow donors, χ^2^(2) = 11.500, p = 0.021 and χ^2^(2) = 11.367, p = 0.023, respectively. (**a**, **b** inlays) A Pearson correlation analysis confirmed this primary role of donor-related factors in the induction variation by indicating a strong correlation between IFNγ-induced upregulation of CD274 and IDO between Flask- and Scinus-expanded MSCs (R^2^ = 0.78 and 0.93 respectively). **c** MSC capacity to inhibit T cell proliferation. **d** Heatmap and cluster analysis of T cell proliferation

Upon stimulation with IFNγ, MSCs have been described to up-regulate various molecules implicated in MSC-mediated immunomodulation [14], including CD274 (PD-L1) and indolamine-pyrrole 2,3-dioxygenase (IDO) that are both involved in dampening T cell mediated immune responses [15–18]. The flask-, spinner- and Scinus-cultured cells were investigated for their ability to upregulate CD274 and IDO protein expression upon stimulation with IFNγ. Both CD274 and IDO expression were found to be induced by IFNγ treatment in flask-, spinner- and Scinus-cultured MSCs (Fig. 5a, b). A Friedman test showed no statistical difference in levels of both CD274 and IDO induction between the culture methods (χ^2^(2) = 1.600, *p* = 0.449 and χ^2^(2) = 2.333, p = 0.311 respectively). To further substantiate that such a potential difference between culture methods was not missed due to measurement inaccuracy a Pearson correlation analysis was performed on the small data set (and assuming a normal data distribution). This indicated a strong and visual correlation between CD274 and IDO induction levels in MSCs expanded using the Scinus and using culture flasks (R^2^ = 0.78 and 0.93 respectively, inlays in Fig. 4a, b). Such correlation indicates that inherent donor-related factors mainly contribute to the functional differences between samples rather than the culture method that was used for expanding the cells.

#### Inhibition of T cell proliferation

The immunomodulatory capacity of the MSC populations generated with the three culture methods was tested using an in vitro T cell proliferation assay, in which PBMCs were stimulated with anti-human CD3/CD28 beads in the presence of varying doses of MSCs. The capacity of the MSCs to inhibit T cell proliferation was similar for all culture methods (Fig. 5c). Accordingly, the Pearson correlation average-linkage analysis showed no hierarchical clustering by culture method (Fig. 5d). Together, we observed no functional differences in MSC-mediated inhibition of T cell proliferation between MSCs that were expanded using the three culturing methods.

## Discussion

In this paper we compared key properties of MSC populations generated with three different culture methods, including a novel closed bioreactor system. The goal was to investigate the potential of the bioreactor for future scale-out of MSC production. For successful scale-out of CTMP manufacturing the following aspects are of importance: (1) (high) cell numbers are reliably obtained, (2) product quality is assured and parity is maintained compared to the original process, and (3) production is efficient. Our comparative study shows evidence that the microcarrier-based bioreactor system meets at least the first two criteria. MSCs were expanded in the bioreactor to therapeutically relevant cell numbers and the resulting MSC populations demonstrated equality to MSC populations generated with a conventional flask-based process with respect to identity, safety and immunomodulatory properties.

A novel clinical expansion method has to reliably achieve clinically relevant cell numbers. In many current clinical MSC applications 1–2 × 10^6^ cells per kilogram recipient body weight are infused. We therefore required that a minimum of 200 × 10^6^ cells was reliably achieved. This minimum was reached in all bioreactor cultures (range 222–510 million). As the cells were still steadily expanding at the day of harvest, cell yield might be increased with prolonged cell culture. Factors that will ultimately limit the maximum yield are the maximum concentration of microcarriers, which imposes the maximum available surface area, and the maximum perfusion speed, which determines the rate of nutrient delivery and waste removal. It should also be noted that the donor material is obtained from relatively old patients (average age 65.4 years) with a diseased joint. While these donors were chosen because they represent an easily accessible donor pool with limited ethical restrictions, they are not necessarily representative of the donor populations used for clinical applications, especially in allogeneic therapies where MSCs are likely obtained from younger and healthy donors.

Continuous growth and high cell numbers were achieved using both microcarrier-based methods, but traditional flask-based culture displayed slightly faster growth. It has been described that expansion of MSCs on microcarriers results in a prolonged lag phase compared to monolayer culture [19, 20], which could have attributed to the observed difference in PDL. Another potential explanation for the difference in observed PDL is the lower seeding density that was used for monolayer culture (400 cells/cm^2^ instead of 1000 cells/cm^2^) to prevent over confluence at the time of harvest. Seeding density can have a measurable effect on growth kinetics, with lower seeding densities resulting in faster proliferation [21]. Interestingly, the microcarrier culture in the Scinus bioreactor resulted in faster growth than culture in the spinners. This difference is most likely attributed to the continuously controlled culture environment in the bioreactor (maintaining pre-set values for DO, pH and temperature). Also, shear forces in the Scinus system are low compared to impeller-based bioreactors (manuscript submitted) and MSCs are known to be sensitive to shear stress, which can affect population doubling times [22].

A recognised issue in microcarrier-based cell expansion is aggregate formation, which may compromise bead-to-bead transfer which is important in the cell expansion phase. Microcarrier aggregates may also impair live cell harvest efficiency in the downstream processing. In this study we obtained single cell suspensions at the end of culture, indicating that harvest efficiency was not compromised. Minimal aggregation was observed which, combined with a straightforward harvesting procedure, resulted in easy cell collection.

MSCs have been defined by the presence and absence of a specific set of surface markers, their ability to adhere to plastic and a tri-lineage differentiation potential, which is based on MSC expansion in tissue culture flasks [23]. MSCs expanded using the three culture methods were morphologically similar, adhered to plastic and expressed CD73, CD90 and CD105, and maintained their differentiation potential in accordance with these criteria. The cells did not display a method-related qualitative difference in differentiation capacity between culture conditions. We demonstrated robust differentiation along the chondrogenic and adipogenic lineage for all donors and all conditions. Osteogenic differentiation was only clearly observed for Donor 1. Other donors displayed minimal to no osteogenic differentiation, but again no difference was observed between the culture conditions. Although expression of CD73 and CD90 was similar between the culture methods, individual cell preparations harvested from the Scinus bioreactor displayed a reduced frequency of CD105^+^ cells. Reduced expression of CD105 in bioreactor cultures has been reported before [24, 25]. We demonstrated that CD105 is susceptible to degradation by the enzyme used in the harvesting process, explaining the lower CD105 expression levels. Indeed, CD105 expression levels were restored after subsequent expansion and harvesting from flasks (data not shown). Thus far, there is no clinical relevance to the expression of CD73, CD90 and CD105 on MSCs, and the expression has rather been used as a characteristic phenotype of the cells. Therefore, it seems arguable to adhere to this criterion to define MSCs. The MSC criteria previously defined have served a good purpose in the flask-based expansion era, but need to be revisited now that more efficient selection and expansion methods become available.

The cell populations cultured with either method did not show differences with respect to the fraction or composition of cellular impurities. Also the karyotypic analyses did not show a compromised genetic profile for either of the culture methods. Therefore, the data collected in this comparative study, indicate a comparable purity and safety profile for the three production methods.

MSCs cultured with the bioreactor system showed equality to flask-expanded cells with respect to their immunomodulatory properties. IFNγ-induced expression of CD274 (PD-L1) and IDO, molecules that are associated with functional inhibition of immune responses [15, 26], was observed for all cultures. Previously, inter-donor variation in the levels of such upregulation has been reported [27]. This phenomenon was also observed in our study, both in the flask cultures as well as in the microcarrier-based cultures. Our IFNγ-induced immunomodulatory molecule expression data indicate that attributes of MSC populations are more influenced by the starting material than by the culture method and indicate parity between the MSC populations resulting from flask and microcarrier-based culture methods.

For logistical reasons our IFNγ-stimulation assay was preceded by an extra plating and culturing step on a flat surface. Although all induction attributes were therefore measured on flat surface-expanded cells, our results indicate that the preceding expansion on microcarriers does not lead to irreversible changes, whereas donor-dependent differences seem to be preserved.

Inhibition of T cell proliferation is the most commonly tested MSC function relevant for the proposed mechanisms of action of MSCs in various clinical trials aiming at immunoregulation [28]. The inter-donor variation in IFNγ-induced expression levels did not translate into significantly different functionality in an in vitro assay measuring the capacity to inhibit T cell proliferation. Our data, therefore, do not support a direct link between responsiveness of CD274 or IDO expression to IFNγ and the capacity to inhibit T cell proliferation.

Manufacturing efficiency is the third crucial aspect for the viability of cell therapies as a whole. Previous pioneering therapies involving expensive production processes have failed due to unfavorable cost–benefit assessments and lack of reimbursement [29]. Therefore, there is a clear need for alternatives to traditional labor-intensive flask-based cell expansion processes. The use of closed, automated and controlled devices is seen as an important step that facilitates affordable cell expansion within GMP regulations [30]. The use of such systems has several benefits: (1) a closed system minimizes the risk of contamination, providing options to ultimately manufacture CTMPs outside of stringently controlled and expensive cleanroom class B environments, (2) the automation reduces labor costs and process variability [25], (3) the controlled environment within the device provides more accurate information about the state of the cell cultures. This information can be used to improve and further standardize the expansion process. All these factors will lead to more robust production processes at reduced costs. We initiated cultures with pre-cultured cells to obtain sufficient cells to seed all conditions and we chose 20 million cells as a starting inoculum since this amount was previously used as an inoculum for other bioreactor studies [25, 31]. The total cell numbers achieved in our study (average 324 million cells) would require an equivalent of 130 T175 flasks, an amount that requires multiple operators to refresh and harvest in a realistic time-frame. In contrast, the total volume of the bioreactor can be refreshed and harvested by one operator within 10 min and 1 h respectively. An interesting feature of the Scinus bioreactor is the option to culture at any volume between 100 to 1400 mL or to increase the volume of the bioreactor bag during the process. This allows the seeding of very low cell numbers at manufacturing start. Currently, protocols using this feature are being optimised to generate clinically relevant MSC numbers from a single BM biopsy. In these protocols the culture volume is gradually increased and MSC colonise freshly added microcarriers by bead-to-bead transfer [32, 33]. These versatile properties make the medium-sized Scinus bioreactor particularly suited for production scale-out.

## Conclusion

We demonstrated that a new microcarrier-based bioreactor system fulfils essential requirements to consider its integration in the further development and scale-up of flask-based MSC manufacturing methods. This system allows MSC expansion to clinically relevant cell numbers, and the resulting cell populations are similar in identity, purity and potency to MSCs expanded in traditional flasks. The system has the potential to efficiently scale-out the production of MSCs for clinical applications.

## Additional file

**Additional file 1.** Differentiation assay results. Results of the tri-lineage differentiation assay for available samples of all donors.

## Data Availability

The datasets used and/or analysed during the current study are available from the corresponding author on reasonable request.

## Abbreviations

hMSC: (human) mesenchymal stromal cell
PDL: population doubling level
IFNγ: interferon-gamma
CTMP: cell therapy medicinal product
GMP: good manufacturing practice
DO: dissolved oxygen
PBS: phosphate buffered saline
MNC: mononuclear cell
FBS: fetal bovine serum
bFGF: basic fibroblast growth factor
DMSO: dimethylsulfoxide
DMC: dissolvable microcarrier
EDTA: ethylenediaminetetraacetic acid
PBMCs: peripheral blood mononuclear cells
IDO: indoleamine 2,3-dioxygenase
MFI: median fluorescence intensity
