# Supplementary material for: Preparing for cell culture scale-out: establishing parity of bioreactor- and flask-expanded mesenchymal stromal cell cultures
Source: J Transl Med. 2019 Jul 24;17:241. doi: 10.1186/s12967-019-1989-x (PMC6657181; doi:10.1186/s12967-019-1989-x)
Supplement: Supplementary file 1 — Additional file 1. Differentiation assay results. Results of the tri-lineage differentiation assay for available samples of all donors. [file 12967_2019_1989_MOESM1_ESM.pptx]

## Slide 1
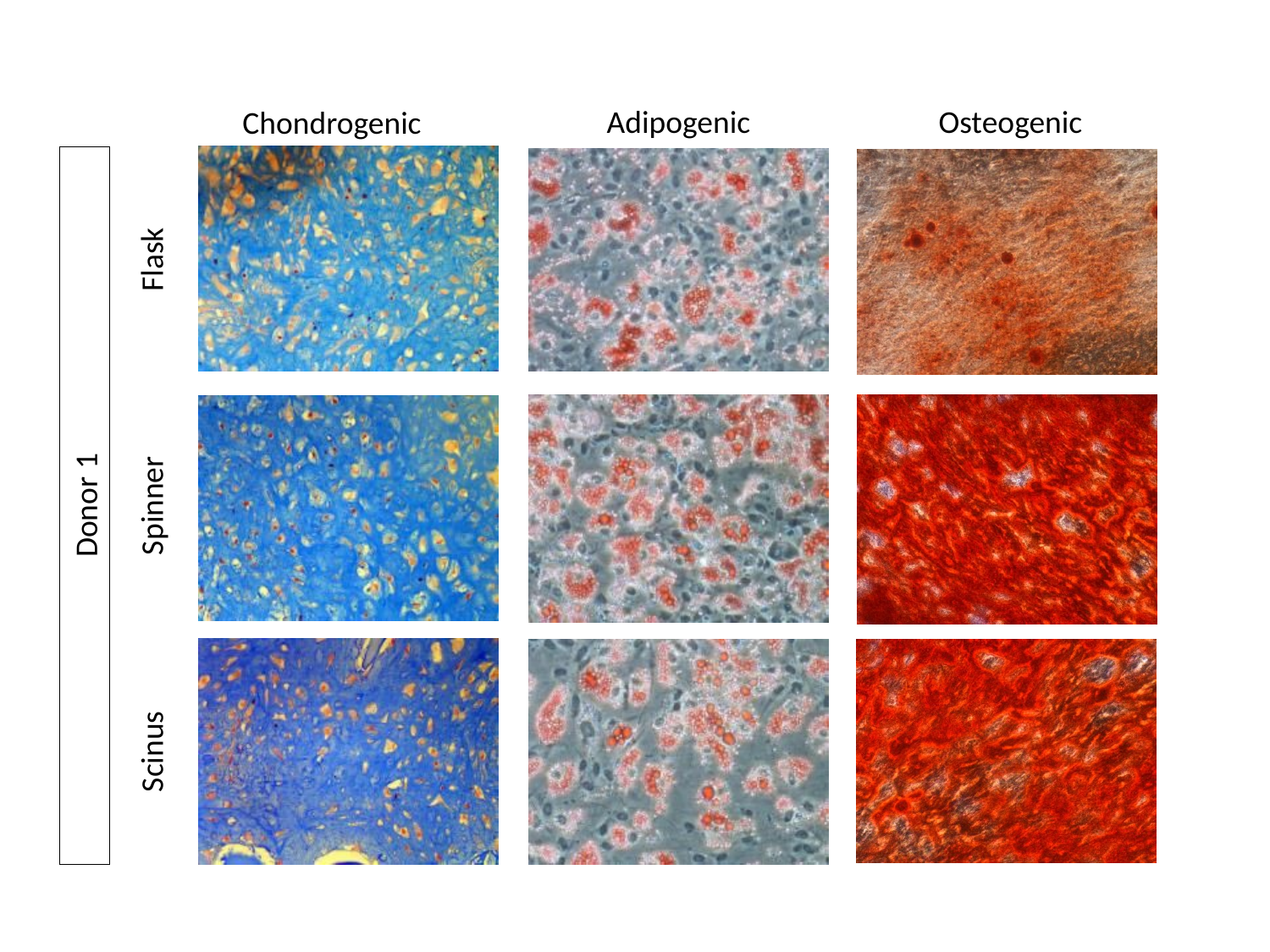

Adipogenic
Osteogenic
Chondrogenic
Flask
Spinner
Scinus
Donor 1

## Slide 2
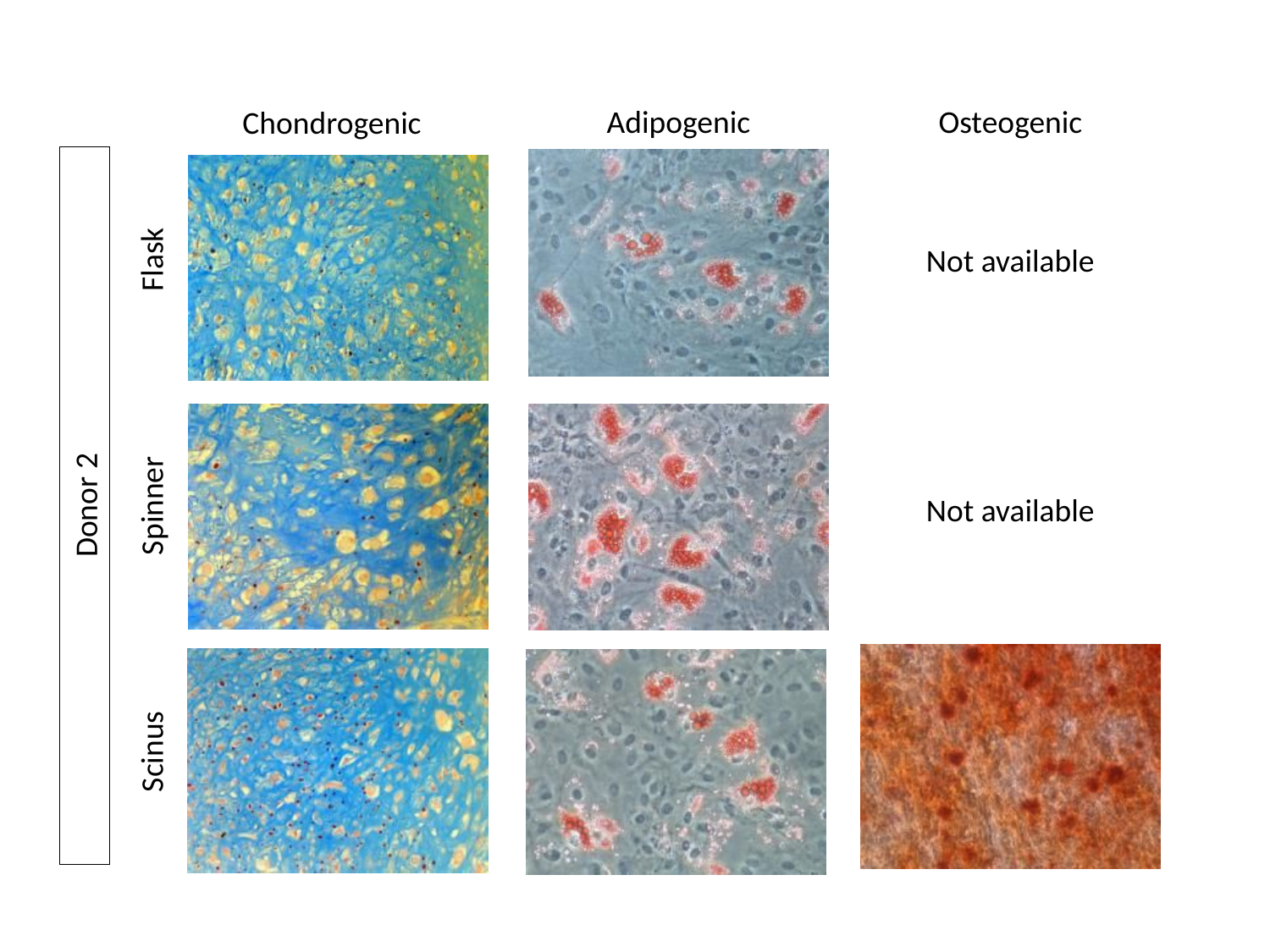

Adipogenic
Osteogenic
Chondrogenic
Flask
Spinner
Scinus
Not available
Donor 2
Not available

## Slide 3
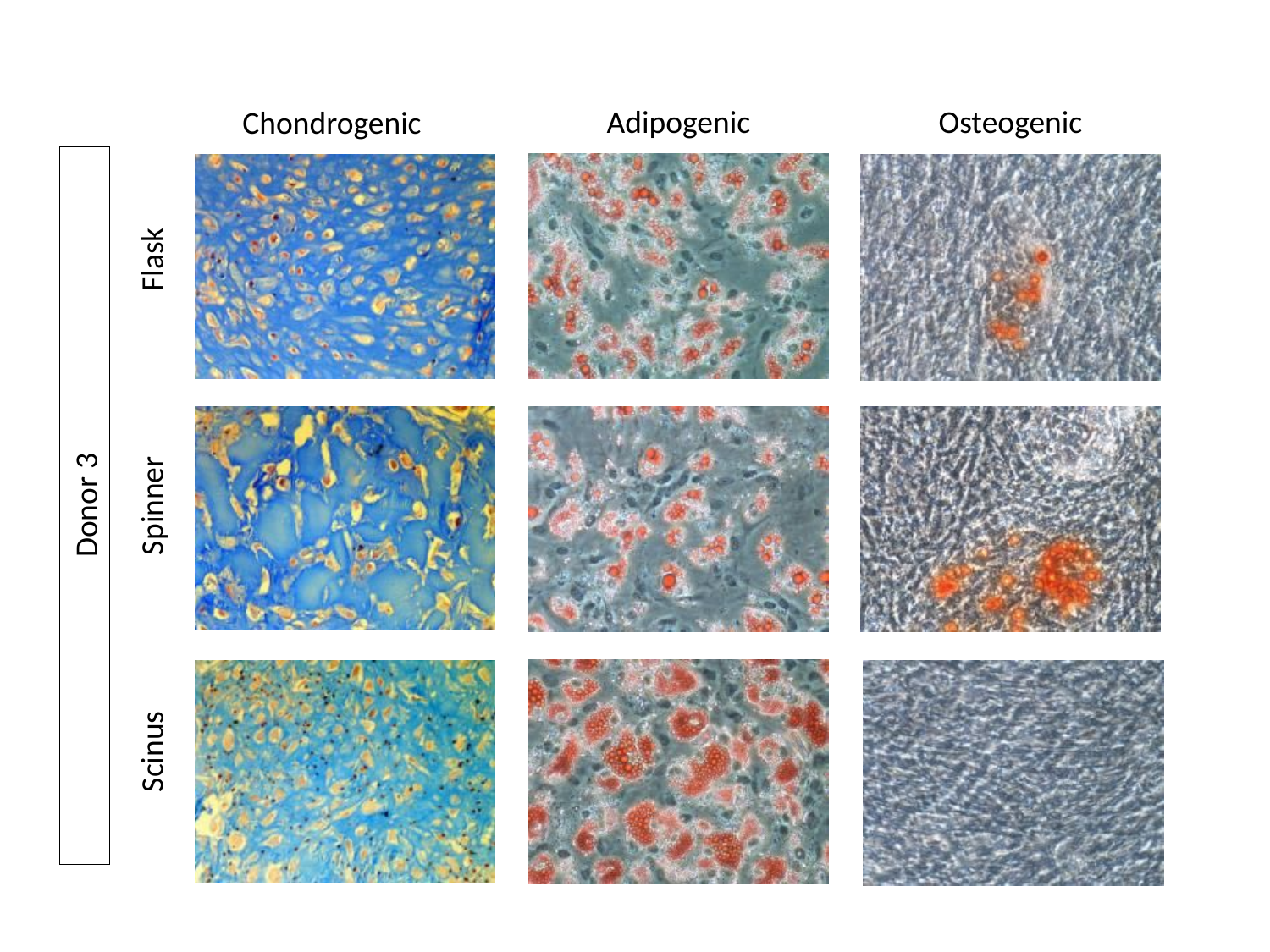

Adipogenic
Osteogenic
Chondrogenic
Flask
Spinner
Scinus
Donor 3

## Slide 4
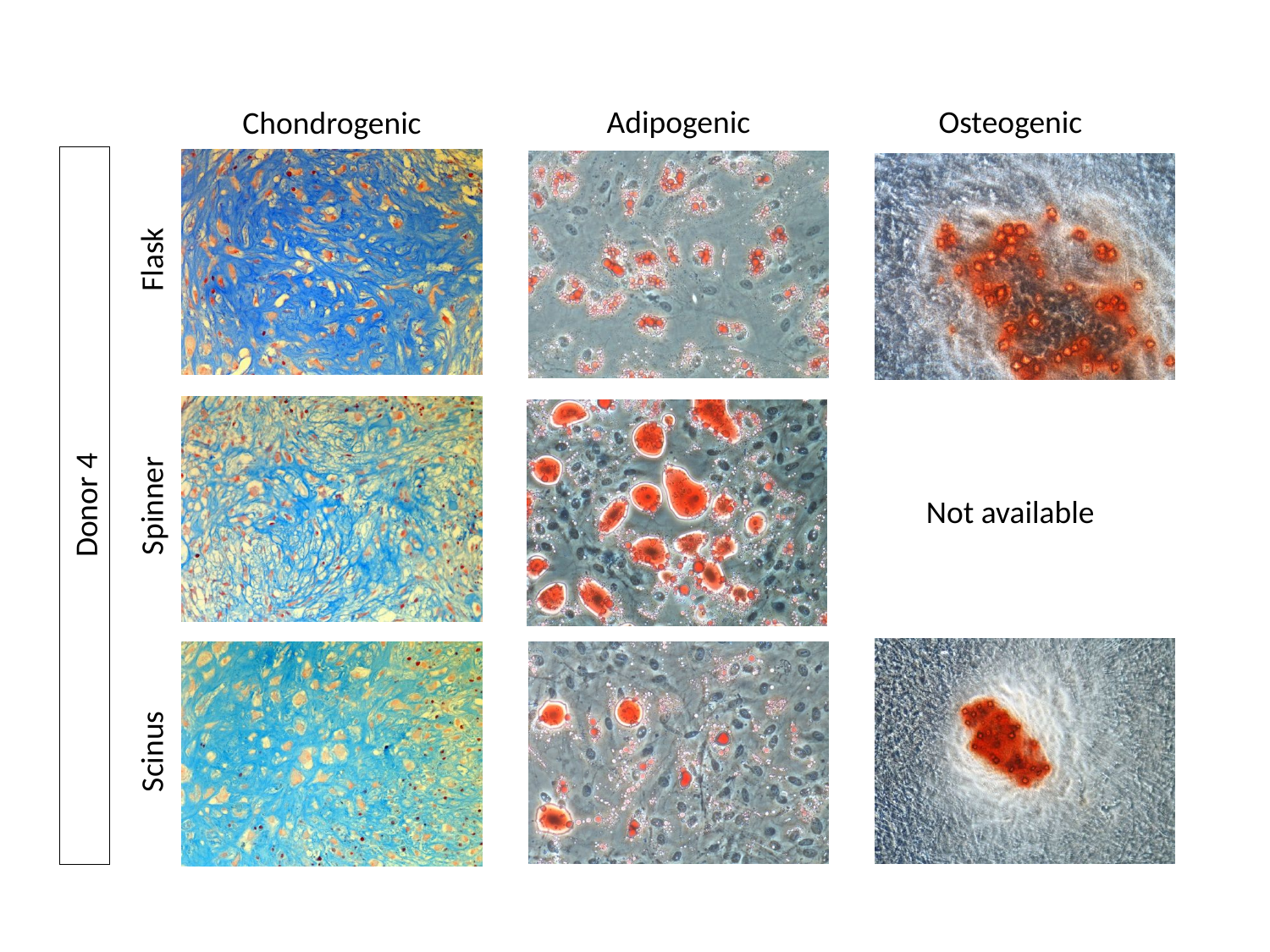

Adipogenic
Osteogenic
Chondrogenic
Flask
Spinner
Scinus
Donor 4
Not available

## Slide 5
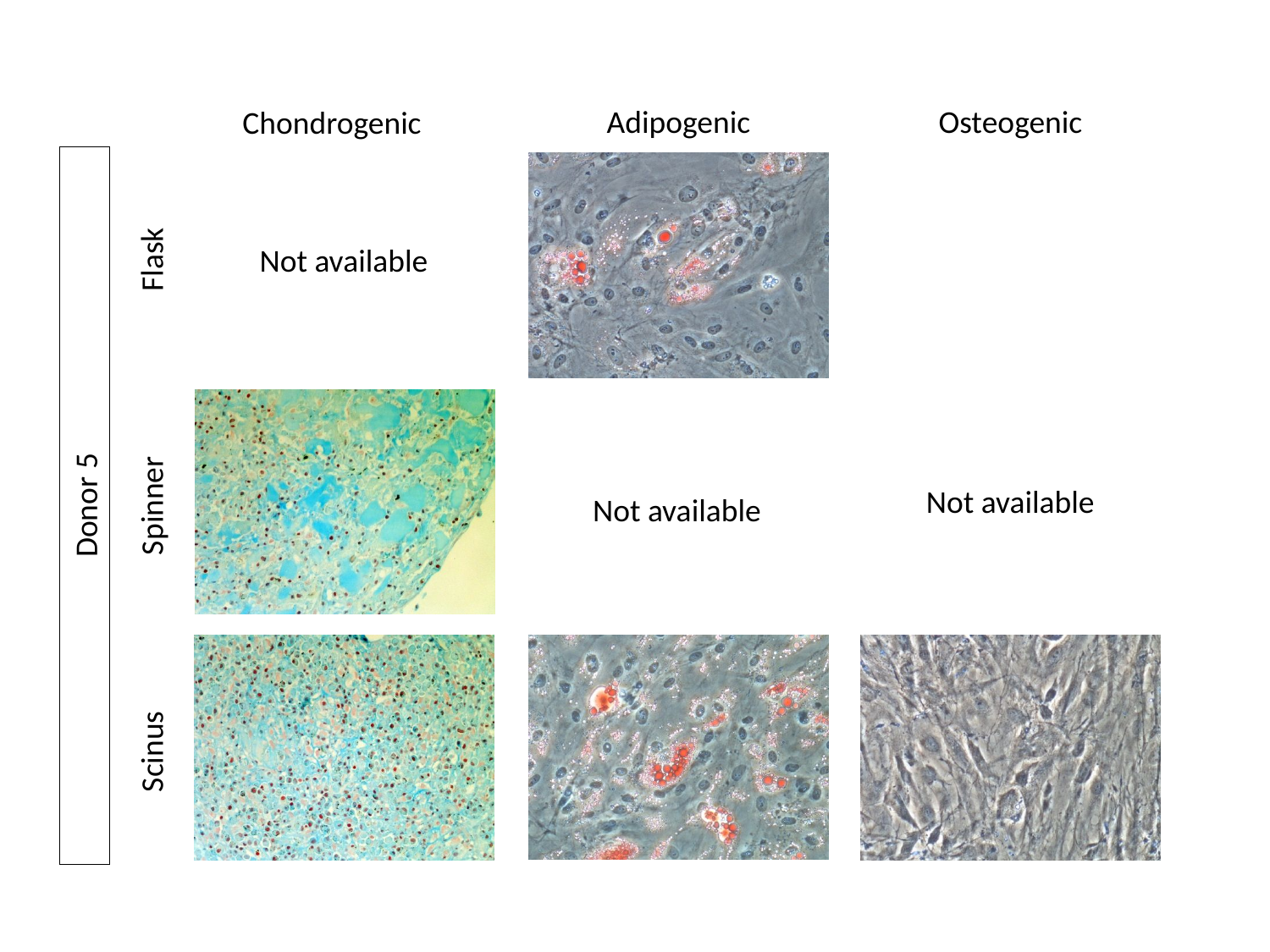

Adipogenic
Osteogenic
Chondrogenic
Flask
Spinner
Scinus
Not available
Not available
Donor 5
Not available
